# In Silico Investigations on the Synergistic Binding Mechanism of Functional Compounds with Beta-Lactoglobulin

**DOI:** 10.3390/molecules29050956

**Published:** 2024-02-22

**Authors:** Tong Meng, Zhiguo Wang, Hao Zhang, Zhen Zhao, Wanlin Huang, Liucheng Xu, Min Liu, Jun Li, Hui Yan

**Affiliations:** 1 School of Pharmaceutical Sciences, Liaocheng University, Liaocheng 252059, China; mengtong19950613@sina.com (T.M.); 15163592080@163.com (H.Z.); 18354060812@163.com (Z.Z.); lijun1982@lcu.edu.cn (J.L.); 2 Institute of Ageing Research, School of Basic Medical Sciences, Hangzhou Normal University, Hangzhou 311121, China; 3 School of Basic Medical Sciences, Hangzhou Normal University, Hangzhou 311121, China; 2020211301217@stu.hznu.edu.cn (W.H.); 2021211301175@stu.hznu.edu.cn (L.X.); 4 School of Chemistry and Chemical Engineering, Liaocheng University, Liaocheng 252059, China; panpanliumin@163.com

**Keywords:** polyphenols, palmitate, β-lactoglobulin, molecular docking, molecular dynamics, MM/GBSA

## Abstract

Piceatannol (PIC) and epigallocatechin gallate (EGCG) are polyphenolic compounds with applications in the treatment of various diseases such as cancer, but their stability is poor. β-lactoglobulin (β-LG) is a natural carrier that provides a protective effect to small molecule compounds and thus improves their stability. To elucidate the mechanism of action of EGCG, PIC, and palmitate (PLM) in binding to β-LG individually and jointly, this study applied molecular docking and molecular dynamics simulations combined with in-depth analyses including noncovalent interaction (NCI) and binding free energy to investigate the binding characteristics between β-LG and compounds of PIC, EGCG, and PLM. Simulations on the binary complexes of β-LG + PIC, β-LG + EGCG, and β-LG + PLM and ternary complexes of (β-LG + PLM) + PIC, (β-LG + PLM) + EGCG, β-LG + PIC) + EGCG, and (β-LG + EGCG) + PIC were performed for comparison and characterizing the interactions between binding compounds. The results demonstrated that the co-bound PIC and EGCG showed non-beneficial effects on each other. However, the centrally located PLM was revealed to be able to adjust the binding conformation of PIC, which led to the increase in binding affinity with β-LG, thus showing a synergistic effect on the co-bound PIC. The current study of β-LG co-encapsulated PLM and PIC provides a theoretical basis and research suggestions for improving the stability of polyphenols.

## 1. Introduction

**Figure 1:**
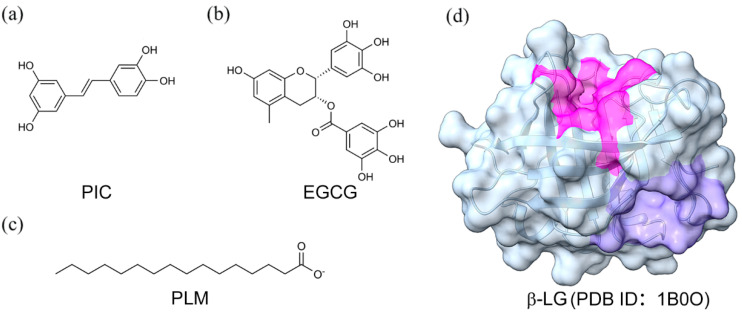
Schematic diagram of PIC (**a**), EGCG (**b**), and PLM (**c**) together with the structure of β-lactoglobulin (**d**). The central and peripheral binding pockets are outlined by magenta and slate blue surfaces, respectively (**d**).

Polyphenols are widely found in plants and have many biological functions and health benefits. Epigallocatechin gallate (Figure 1a), which is one of the most common polyphenols, can effectively inhibit the growth and induce apoptosis in human breast cancer cells [1,2,3,4], lung cancer cells [5], prostate cancer cells [6], etc. Piceatannol (Figure 1b) is a hydroxylated analog of resveratrol. Many studies have shown that PIC is a potent inhibitor of apoptosis in lymphoma cell lines [7] and primary leukemia cells [8]. However, polyphenolic compounds contain multiple phenolic hydroxyl groups in their structures, which leads to their reduced pH/light stability and photosensitivity [9]. Protection of polyphenolic compounds can be achieved by co-coating them with proteins, in which lactoglobulin is a natural delivery carrier that binds to small molecules to form protein–ligand complexes, thereby protecting polyphenolic compounds [10,11,12]. Palmitate (PLM, Figure 1c) is a medium- to long-chain fatty acid found in saturated fat-containing foods with potential benefits in skin health, anti-inflammation, and metabolism enhancement [13].

In addition, there are synergistic effects among the compounds when proteins encapsulate two or more polyphenols. Studies have shown that epigallocatechin gallate is more biologically active and stable when administered in combination with other biologically active ingredients than when administered as a single substance [14]. The antioxidant properties of polyphenols and palmitate are well studied, but using polyphenols and polyphenol/palmitate combinations in conjunction with proteins is relatively understudied and warrants further research.

Direct observation of these microscopic interactions is challenging due to limitations in experimental techniques. Therefore, investigating these interactions between polyphenols and proteins through computational methods can provide theoretical insights into the microscopic mechanism for protein drug carriers, which is essential for further developing polyphenol-based anticancer drugs. Over the past decades, molecular dynamics (MD) simulations have proven to be a powerful technique that can provide complementary and microscopic insights into experimental observations. Many computational studies have attempted to gain insight into the microscopic behavior of polyphenol molecules interacting with proteins. Kinetic studies related to the binding of proteins by a single EGCG small molecule have been reported [15]. However, studies on the molecular dynamics of EGCG and PIC co-binding to proteins have not been reported, especially the cooperative interactions among drug molecules bound to protein carriers, which deserve further investigation. 

In our recent study [16], we used beta-lactoglobulin (β-LG, Figure 1d) as a carrier to encapsulate EGCG, PIC, and oxidized resveratrol (OXY) and we investigated the effects of ligand–protein binding on these three active ingredients’ antioxidant activity, stability, solubility, and cytotoxicity. Stability and solubility experiments showed that the addition of β-LG significantly improved the stability and solubility of the three polyphenols. β-LG, one of the most widely studied food proteins, plays a vital role in the milk of mammals. It is rich in nutrients and has multiple functional properties, making it an ideal vehicle to load a variety of natural active ingredients, which can achieve the protection of polyphenol function [17] and provide multiple health benefits. Therefore, in-depth studies on the interaction mechanism between proteins and polyphenols and the effect of palmitic acid (PLM) on the complexes between proteins and polyphenols are essential for developing effective polyphenolic anticancer drugs.

In this work, by performing molecular docking and explicit molecular dynamics simulations, we revealed the mechanism of action between polyphenol compounds and β-LG through the characterization of root-mean-square deviation (RMSD), root-mean-square fluctuation (RMSF), noncovalent interactions (NCI), principal component analysis (PCA), dynamic cross-correlation (DCC), and binding free energies, which will provide theoretical bases for improving the further research and development of polyphenol compounds.

## 2. Results and Discussion

### 2.1. Binding Modes of PLM, EGCG, and PIC with β-LG

The β-LG crystal structure retrieved from the protein data bank is a binding complex of β-LG + PLM (PDB ID: 1B0O). We first redocked PLM into β-LG to verify the accuracy of the docking method. The PLM conformation obtained by molecular docking was well superimposed with the crystal form (Figure S1), validating the accuracy of the current docking method.

**Figure 2:**
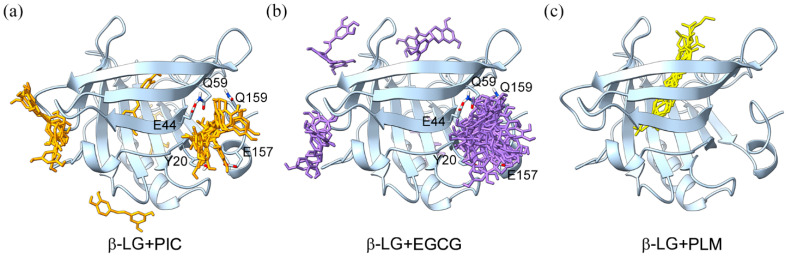
Docking predicted binding conformations of PIC (**a**), EGCG (**b**), and PLM (**c**) with β-LG. The molecules of PIC, EGCG, and PLM are colored in orange, purple, and yellow, respectively.

We docked EGCG and PIC to β-LG separately and exported twenty possible binding poses for each compound (Figure 2). Though three to four potential binding sites were predicted for both EGCG and PIC, the peripheral pocket defined by Y20, E44, Q59, E157, Q159, and adjacent residues should be most probable since docked poses were most populated here and showed advantages in binding affinity (Figure 2a,b). The identified binding mode is consistent with previous reports. Specifically, Kanakis et al. found that EGCG binds to the sidewalls of the β-barrel structure of β-LG by spectroscopic and molecular docking studies [18]. Liu Min’s team revealed that the PLM located at the central pocket of β-LG showed neglectable effects on the binding of EGCG and the PIC [16], further strengthening the reliability of our docking calculations. For compound PLM, all the docked poses were located at the center of β-LG and showed a good superimposition with the crystal configuration (Figure 2c and Figure S1).

The docked poses of PIC, EGCG, and PLM molecules were ranked by the binding energies, and the root-mean-square deviation (RMSD) values relative to the first pose that showed the highest binding affinity were listed to show the conformational differences among all poses (Tables S1–S3). For PIC and EGCG, the poses that showed the most negative binding energies were recognized as potent bioactive binding conformations and were subjected to subsequent MD simulations to characterize their dynamic binding features with β-LG. For PLM, the crystal configuration was directly used (Figure S2). Moreover, Amber score [19], a physics-based scoring function embedded in Dock6 [20], was applied to re-rank the top ten poses from the Vina calculation. Two additional poses of PIC and EGCG that performed best under the Amber score were submitted to MD simulations as well, in order to investigate the effect of initial structures on the convergence of the equilibrated states. For PLM, the additional MD simulations were still based on the crystal structure.

To investigate the interactions between the bound polyphenol compounds, a molecule of EGCG/PIC was further docked to the β-LG + PIC/EGCG binary complex (Figure S3) to form the corresponding ternary binding complexes, which were denoted as (β-LG + PIC) + EGCG and (β-LG + EGCG) + PIC, respectively. Interestingly, both EGCG and PIC were found to prefer binding to the β-barrel cavity (Figure S3). The ternary binding complexes of (β-LG + PLM) + PIC and (β-LG + PLM) + EGCG were constructed by incorporating the crystal configuration of PLM and the best-scored poses of EGCG and PIC (Vina score), respectively (Figure S3). Similarly, two additional MD simulations based on different starting structures were performed for each ternary binding complex.

### 2.2. Binding Characteristics of the β-LG and Compounds of PIC, EGCG, and PLM

**Figure 3:**
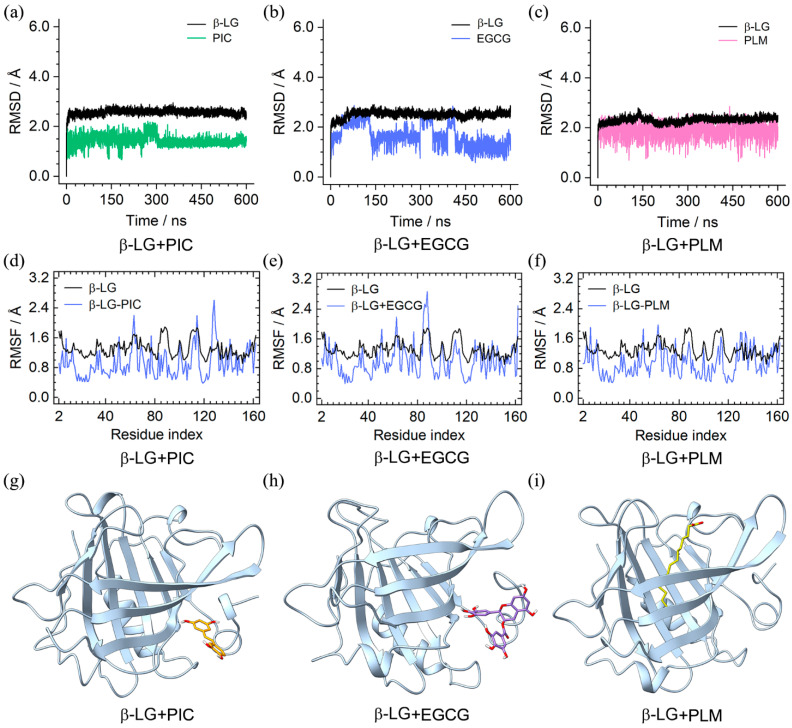
MD simulations of the binary complexes of β-LG + PIC/EGCG/PLM. (**a**–**c**) RMSD profiles of β-LG and the bound compounds; (**d**–**f**) root-mean-square fluctuations (RMSFs) of the compound-bound β-LG; (**g**–**i**) equilibrated binding conformations of the binary complexes. The molecules of PIC, EGCG, and PLM are colored in orange, purple, and yellow, respectively.

The RMSD results of the binary complexes indicated that β-LG and the binding compounds of PIC, EGCG, and PLM achieved equilibrium within 600 ns (Figure 3a–c). The RMSF profiles of β-LG that were calculated based on the MD trajectories showed a consistent fluctuation pattern with the one that was converted from the PDB B-factors (Figure 3d–f), enhancing the credibility of our MD simulations. Observations on the MD-equilibrated conformations of the binary complexes found that, in spite of some orientational alternations, the bound PIC, EGCG, and PLM remained located at their initial binding sites (Figure 3g–i and Figure S2), indicating their high binding stability with β-LG.

**Figure 4:**
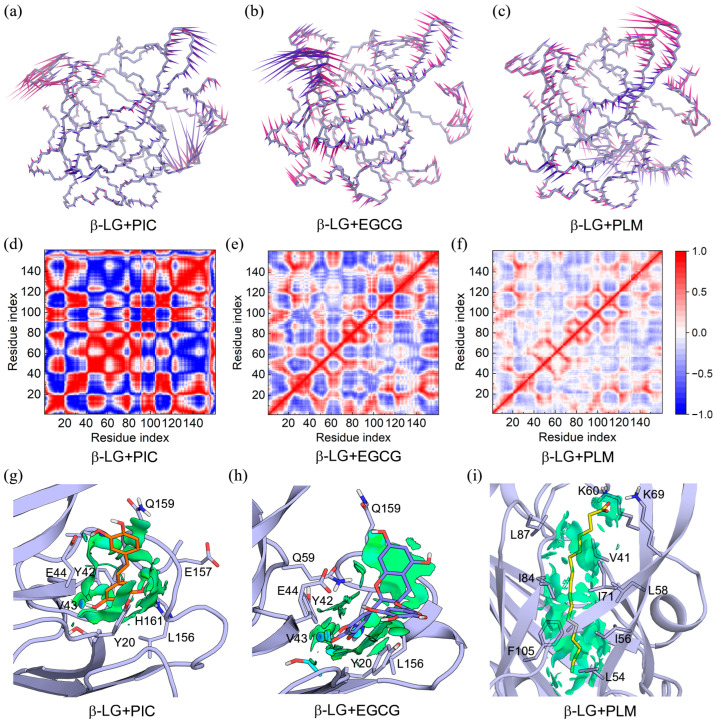
The dynamics feature of the PIC/EGCG/PLM-bound β-LG and their intermolecular interactions. (**a**–**c**) Porcupine plots of the PIC/EGCG/PLM-bound β-LG, with the first and second eigenvectors colored in violet and magenta, respectively; (**d**–**f**) dynamic cross-correlation map for the Cα atom pairs within the PIC/EGCG/PLM-bound β-LG. Correlation coefficients are shown as different colors, with values from 0 to 1 representing positive correlations, whereas values from −1 to 0 represent negative correlations; (**g**–**i**) noncovalent interactions between β-LG and the binding compounds (isovalue of 0.3 au). The green and blue isosurfaces indicate the vdW and hydrogen bond interactions, respectively.

PCA calculation showed that over 20% of essential motions can be characterized by the first two eigenvectors of the PIC/EGCG/PLM-bound β-LG (Figure S4), which were, therefore, represented with a porcupine plot (Figure 4a–c). These two eigenvectors mainly corresponded to the motions of peripheral loop structures, and the central β-barrels were of high stability, as indicated by the small arrows. Notably, the regions presenting large motions are the ones with high RMSF values (Figure 3d–f), mutually verifying the reliability of the two results.

Dynamics cross-correlations between the Cα atom pairs are shown in Figure 4d–f. The pairwise cross-correlation coefficients indicate the extent to which the fluctuation of an atom is correlated or anticorrelated with another atom. For the PIC-bound β-LG, strong positive correlations such as amino acids (aa) 22–40 and aa 100–120, and strong negative correlations such as aa 2–20 and aa 22–40, were identified (Figure 4d). The EGCG and PLM-bound β-LG showed a similar correlation pattern, with the latter being weaker in correlation strength (Figure 4e,f).

In order to accurately identify the close interactions between β-LG and the bound compounds, NCIplot analysis was performed to present the interactions as isosurfaces (Figure 4g–i). All three compounds interacted with β-LG through vdW interactions, as indicated by the extensive green isosurfaces. In addition, both PIC and EGCG formed hydrogen bonds with the main chain of V43, and PLM tended to form hydrogen bonds and electrostatic interactions with K60 and K69.

MD simulations on the additional replicas of β-LG + PIC/EGCG/PLM showed that the equilibrated PIC and EGCG in replica 2 and PLM in both replica 2 and replica 3 superimposed well with the ones in replica 1, indicating the convergence of our main (replica 1) MD simulations (Figure S5). The locational difference of compounds PIC and EGCG in replica 3 is probably due to their over-deviated initial structures relative to those in replica 1 (Figure S2).

### 2.3. Characteristics of the (β-LG + PLM) + PIC/EGCG Ternary Complexes

**Figure 5:**
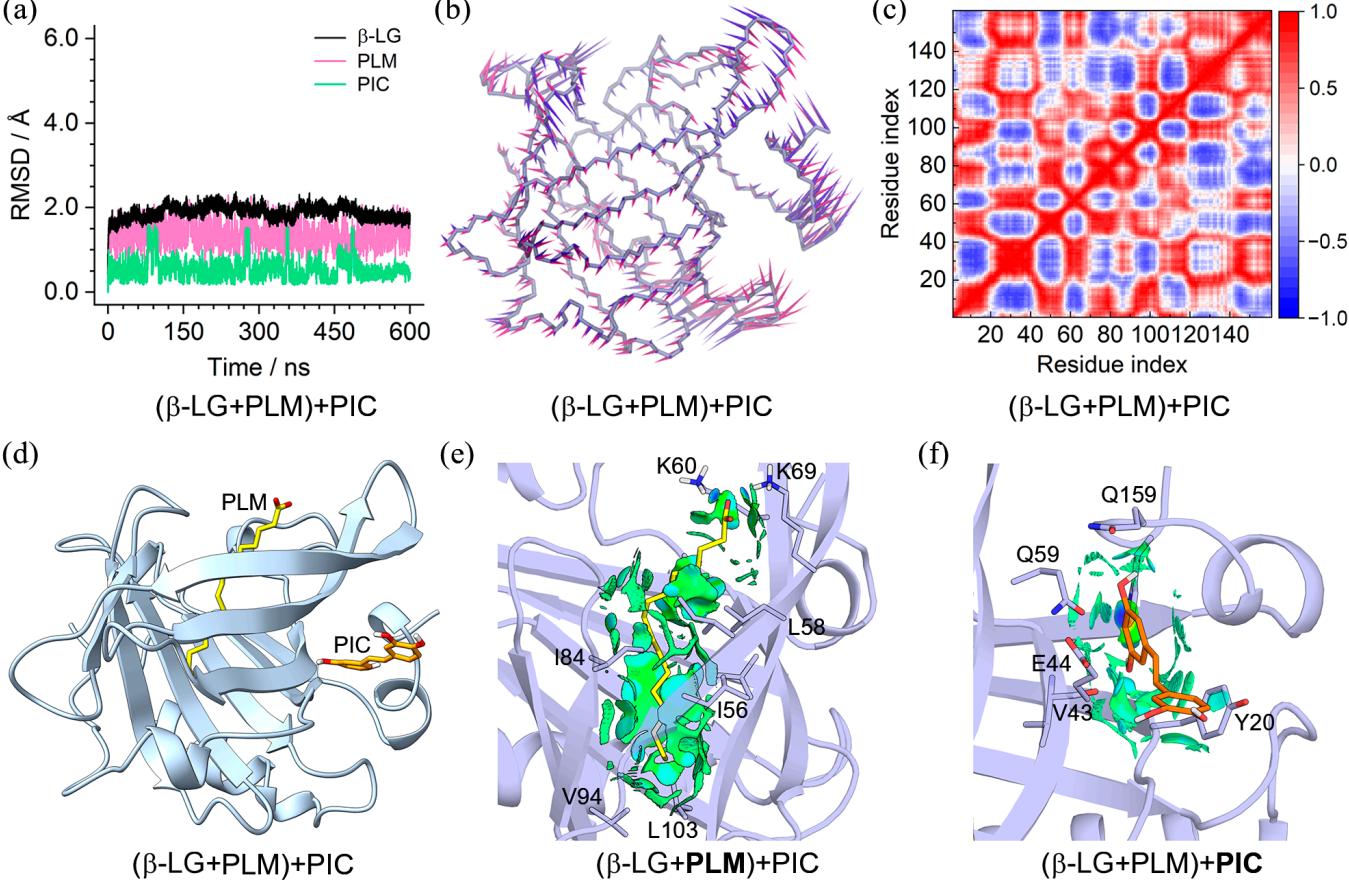
Characteristics of the (β-LG + PLM) + PIC binding complex. (**a**) RMSDs of β-LG and the binding PLM and PIC; (**b**) porcupine plot of the first (violet) and the second (magenta) eigenvectors of the PLM and PIC-bound β-LG; (**c**) dynamic cross-correlation map for the Cα atom pairs within the PLM and PIC-bound β-LG. Correlation coefficients are shown as different colors, with values from 0 to 1 representing positive correlations, whereas values from −1 to 0 represent negative correlations; (**d**) MD-equilibrated binding conformation of (β-LG + PLM) + PIC; (**e**,**f**) NCI surface around PLM and PIC in the binding site of β-LG (isovalue of 0.3 au). The green and blue isosurfaces indicate the vdW and hydrogen bond interactions, respectively.

**Figure 6:**
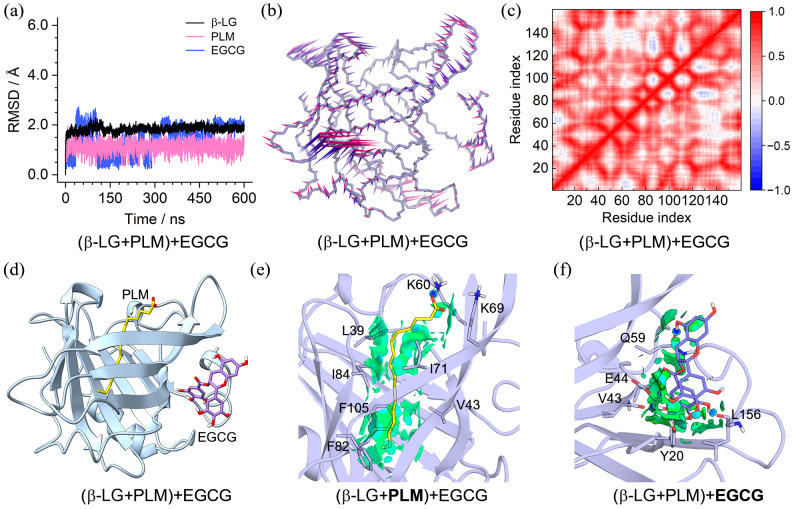
Characteristics of the (β-LG + PLM) + EGCG binding complex. (**a**) RMSDs of β-LG and the binding PLM and EGCG; (**b**) porcupine plot of the first (violet) and the second (magenta) eigenvectors of the PLM and EGCG-bound β-LG; (**c**) dynamic cross-correlation map for the Cα atom pairs within the PLM and EGCG-bound β-LG. Correlation coefficients are shown as different colors, with values from 0 to 1 representing positive correlations, whereas values from −1 to 0 represent negative correlations; (**d**) MD-equilibrated binding conformation of (β-LG + PLM) + EGCG; (**e**,**f**), NCI surface around PLM and EGCG in the binding site of β-LG (isovalue of 0.3 au). The green and blue isosurfaces indicate the vdW and hydrogen bond interactions, respectively.

In order to explore the effect of PLM presence in the β-LG central cavity on the binding of PIC and EGCG, molecular dynamics simulations of the (β-LG + PLM) + PIC and (β-LG + PLM) + EGCG ternary complexes were performed. The converged RMSD profiles in Figure 5a and Figure 6a indicate that both binding complexes achieved MD equilibrium within a 600 ns simulation. Affected by the binding compounds, the RMSF profiles showed obvious deviations at the C-terminal and loop of aa 125–129 for the PLM + PIC and PLM + EGCG-bound β-LG, respectively (Figure S6a,b). PCA revealed that over 20% of essential motions can be characterized by the first two eigenvectors for the PLM + PIC- and PLM + EGCG-bound β-LG (Figure S4d,e). For the PLM + PIC-bound β-LG, major motions happened at the C-terminal and the loop of aa 122–129 (Figure 5b). For the PLM + EGCG-bound β-LG, major motions happened at the loops of aa 47–53, aa 83–89, and aa 102–107 with relatively lower scales (Figure 6b). Correspondingly, for the PLM + PIC and PLM + EGCG-bound β-LG, the DCC maps presented relatively strong (both positive and negative) and weak (mainly positive) correlations within and between the Cα pairs of loop residues, respectively (Figure 5c and Figure 6c).

The equilibrated binding conformations shown in Figure 5d and Figure 6d demonstrate that PLM and PIC/EGCG located at the same binding site as their initial binding and similar cases were found for replica 2 and replica 3 (Figure S7c,f), indicating their converged binding states. A closer observation of the intermolecular interactions through NICplot revealed that the hydrophobic residues of the b-barrel played a key role in the binding of PLM through vdW interactions, with K60 and K69 providing essential contributions as well through hydrogen bond and electrostatic interactions (Figure 5e and Figure 6e). For PIC, vdW interactions with hydrophobic residues such as Y20 and hydrogen bond interaction with H161 were discovered (Figure 5f). For EGCG, more extensive vdW interactions with surrounding hydrophobic residues and hydrogen bond interactions with the main chains of V43 and L156 and the side chains of E44 and Q59 were identified (Figure 6f).

### 2.4. Binding Characteristics of the (β-LG + PIC/EGCG) + EGCG/PIC Ternary Complexes

**Figure 7:**
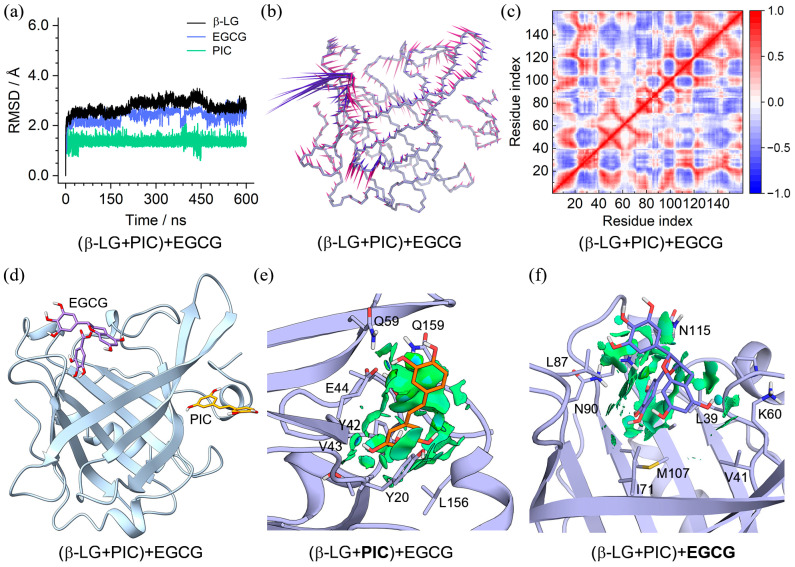
Characteristics of the (β-LG + PIC) + EGCG binding complex. (**a**) RMSDs of β-LG and the binding PIC and EGCG; (**b**) porcupine plot of the first (violet) and the second (magenta) eigenvectors of the PIC and EGCG-bound β-LG; (**c**) dynamic cross-correlation map for the Cα atom pairs within the PIC and EGCG-bound β-LG. Correlation coefficients are shown as different colors, with values from 0 to 1 representing positive correlations, whereas values from −1 to 0 represent negative correlations; (**d**) MD-equilibrated binding conformation of (β-LG + PIC) + EGCG; (**e**,**f**) NCI surface around PIC and EGCG in the binding site of β-LG (isovalue of 0.3 au). The green and blue isosurfaces indicate the vdW and hydrogen bond interactions, respectively.

**Figure 8:**
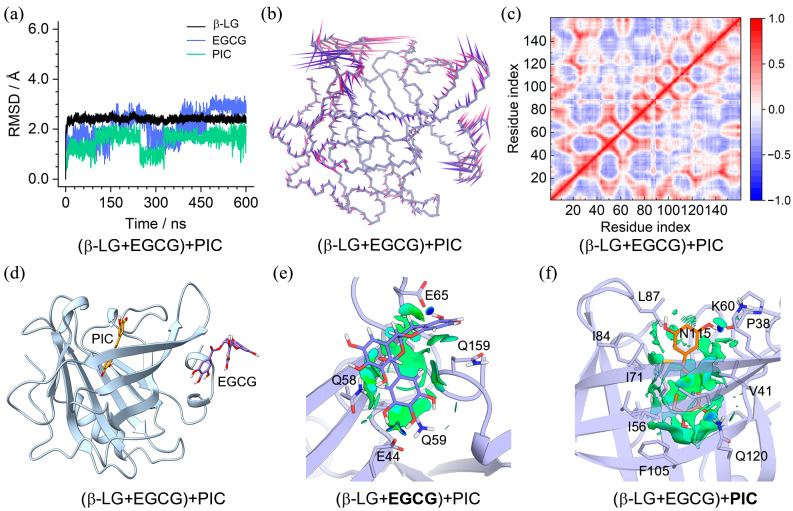
Characteristics of the (β-LG + EGCG) + PIC binding complex. (**a**) RMSDs of β-LG and the binding EGCG and PIC; (**b**) porcupine plot of the first (violet) and the second (magenta) eigenvectors of the EGCG and PIC-bound β-LG; (**c**) dynamic cross-correlation map for the Cα atom pairs within the EGCG and PIC-bound β-LG. Correlation coefficients are shown as different colors, with values from 0 to 1 representing positive correlations, whereas values from −1 to 0 represent negative correlations; (**d**) MD-equilibrated binding conformation of (β-LG + EGCG) + PIC; (**e**,**f**) NCI surface around EGCG and PIC in the binding site of β-LG (isovalue of 0.3 au). The green and blue isosurfaces indicate the vdW and hydrogen bond interactions, respectively.

To investigate the co-binding feature of PIC and EGCG with β-LG, MD simulations were performed on the ternary complexes of (β-LG + PIC) + EGCG and (β-LG + EGCG) + PIC. The results shown in Figure 7 and Figure 8 indicate that both complexes reached an equilibrium state within 600 ns. PCA showed that 56.36% and 29.27% of essential motions of the PIC + EGCG- and EGCG + PIC-bound β-LG can be represented by the first two eigenvectors, with the corresponding motions mainly located at the loop of aa 83–90 ((β-LG + PIC) + EGCG) and at the C-terminal and the loops of aa 83–90 and aa 107–112 ((β-LG + EGCG) + PIC), respectively (Figure S4f,g). The DCC maps shared a similar correlation pattern of Cα pairs, with the ones in (β-LG + PIC) + EGCG presenting slightly higher correlation strength (Figure 7f and Figure 8f). Moreover, the highest RMSF values were discovered at the aa 83–90 in both RMSF profiles (Figure S6c,d), consistent with the PCA result.

The equilibrated binding conformation of (β-LG + PIC) + EGCG shown in Figure 7d and Figure S7i demonstrated a converged binding mode, although EGCG that bound to the central cavity of β-LG exhibited relatively larger conformational variations. PIC in the peripheral pocket showed the same binding mode as in the β-LG + PIC complex (Figure 4g and Figure 7e). However, EGCG in the central site adopted a different binding mode compared to PLM; it can only be located at the upper part of the pocket due to its much larger molecular size (Figure 7f), whereas the equilibrated conformations of (β-LG + EGCG) + PIC shown in Figure 8d and Figure S7l indicate a converged binding mode of PIC and a loose binding of EGCG. EGCG in all replicas is apparently more outward compared to the one in the β-LG + EGCG (Figure 3h and Figure 8d), resulting in more fragmented NCI isosurfaces (Figure 8e). PIC in the central site showed a similar binding mode to PLM, though the binding strength may be probably lowered due to its disadvantage in molecular length (Figure 8f).

### 2.5. Binding Free Energies between β-LG and the Binding Compounds

To evaluate the affinities between β-LG and binding compounds, binding free energies were obtained through molecular mechanics/generalized Born surface (MM/GBSA) calculations. The results in Table 1 indicate that the vdW interaction (ΔE_vdW_) contributed most to the binding in all cases. The contributions from electrostatic interactions (ΔE_ele_) and the polar solvation effects (ΔG_GB_) are always opposite, and the net contributions from ΔE_ele_ and solvation effects (including the polar part ΔG_GB_ and the nonpolar part ΔG_SA_) are small relative to ΔE_vdW_. According to the equation of $∆G_{bind}=\Delta H\;-\;T\Delta S,$ the entropy item always makes unfavorable contributions since the binding led to the decrease in degrees of freedom.

The binding affinities of compounds followed an order of PIC (−10.32 kcal∙mol^−1^) < EGCG (−17.20 kcal∙mol^−1^) < PLM (−19.30 kcal∙mol^−1^) in the binary complexes. Interestingly, PLM binding in the central cavity was found to enhance PIC’s binding affinity to −12.83 kcal∙mol^−1^, and in the meantime, the PLM’s binding affinity remained basically unchanged, presenting a synergistic effect. Such an effect was not observed between the co-bound PLM and EGCG; PLM binding led to the decrease in EGCG’s binding affinity (−15.71 kcal∙mol^−1^), but, conversely, the bound EGCG increased the binding strength of PLM (−21.88 kcal∙mol^−1^). With PIC binding at the peripheral site, EGCG showed a comparable affinity (−17.03 kcal∙mol^−1^) by binding at the central site, indicating that PIC and EGCG may concomitantly bind to β-LG ((β-LG + PIC) + EGCG) without affecting each other’s binding strength. However, PIC binding at the central site would significantly decrease the binding affinity of EGCG (−6.37 kcal∙mol^−1^), indicative of the unfavorable binding mode of (β-LG + EGCG) + PIC.

**Figure 9:**
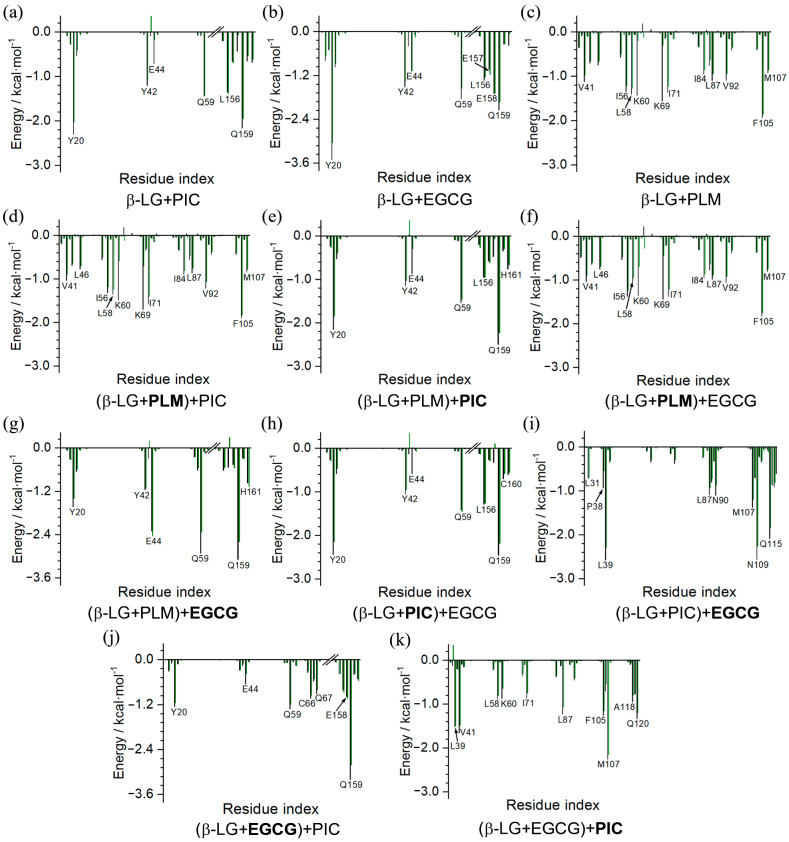
Per-residue decomposition of the binding free energy. (**a**–**k**)The contributions from the vdW interactions and the combined overall contributions are represented by the green and black columns, respectively.

The binding free energies were further decomposed to evaluate per-residue contributions, under conditions in which entropic contributions were excluded. Intriguingly, the per-residue free energy contributions calculated based on the trajectories of the last 100 ns of MD simulations were consistent with the information shown in the NCIplot (Figure 4, Figure 5, Figure 6, Figure 7, Figure 8 and Figure 9), which was based on the MD-equilibrated structures. Moreover, except for the charged residues (such as E44, K60, and K69) that contributed to the binding mainly through electrostatic interactions, the other residues preferred to bind with the compounds mainly through vdW interactions, as indicated by the green columns in Figure 9. The residues that made significant contributions to the binding in the binary and ternary complexes were clearly identified, providing valuable information for the understanding of the protection mechanism of β-LG to functional compounds.

## 3. Methods

### 3.1. Data

The initial structure of β-LG was retrieved from the PDB data bank with the ID of 1B0O [21]. The structures of PIC/EGCG were generated using the GaussView software version 6.0.16 (Gaussian, Wallingford, CT, USA) and were optimized at the DFTB3LYP/6-31G(d) level [22,23]. The atomic partial charges of PIC, EGCG, and PLM were calculated using the restricted electrostatic potential (RESP) method with a basis set of HF/6-31G(d) [24]; the force field parameters of these compounds were then generated using AmberTools (AMBER, San Francisco, CA, USA) [25].

### 3.2. Molecular Docking

Molecular docking calculations were performed using the AutoDock Vina 1.2.5 software [26]. The protonation states of the titratable residues of β-LG were determined with PropKa 3.0 [27] and the compounds of PIC, EGCG, and PLM were prepared with the Auto-DockTools software (version 1.5.6) [28]. The Gasteiger charges were computed for both β-LG and compounds, with the nonpolar hydrogen atoms merged. All the rotatable bonds of compounds were set as flexible, while β-LG was set as the rigid receptor. In each docking calculation, a cubic box centered at the geometric center of β-LG comprising 50 × 50 × 50 grids with a grid spacing of 1.0 Å was used to define the possible binding region. The box was large enough to encompass the whole structure of β-LG so that no binding modes were pre-excluded. The exhaustiveness parameter was set to 17 and all other parameters were set as the default. To construct the ternary binding complexes of (β-LG + PLM) + PIC/EGCG and (β-LG + PIC/EGCG) + EGCG/PIC, the second compound was docked to β-LG with the binding region defined by a cubic box that was just enough to encompass the corresponding pocket.

### 3.3. Molecular Dynamics

Based on the docking-derived binding structures, the binary complexes of β-LG + PIC/EGCG/PLM and the ternary complexes of (β-LG + PLM) + PIC/EGCG and (β-LG + PIC/EGCG) + EGCG/PIC were subjected to MD simulations by using the Amber 20 software [29,30]. Each binding complex was placed in a truncated octahedron box of TIP3P water molecules at a margin distance of 10.0 Å. Environmental Na+ ions were added to maintain electrical neutrality. The Amber ff14SB force field was applied for β-LG. For the compounds, RESP charge together with the second generation of general Amber force field (GAFF2) was applied [31]. Each model was firstly energy minimized by 10,000 steps of steepest descent minimization with a harmonic constraint of 500 kcal∙mol^−1^∙ Å^−2^ imposed on binding complex (solute), followed by 10,000 steps of conjugated gradient minimization with no constraint. Then, the system was gradually heated from 0 to 300 K under the NVT ensemble for 500 ps, with a weak constraint of 10 kcal∙mol^−1^∙ Å^−2^ imposed on the solute. The model was subsequently subjected to an equilibrium simulation for 1 ns by removing all constraints. Finally, the production simulation for each model was conducted under the NPT ensemble with a simulation time of 600 ns (replica 1). To investigate the convergence of MD simulations, two additional replicas based on different starting structures for each model were simulated for 200 ns. In all MD simulations, parameters were set according to our previous report [32]. MD trajectories were recorded at an interval of 10 ps for the structural and energetic analyses.

### 3.4. Principal Component Analysis

Since principal component analysis (PCA) can filter the essential degrees of freedom from a number of local fluctuations [33], PCA was carried out for all MD trajectories using the interactive essential dynamics (IED) method [34]. Based on 5000 frames evenly extracted from the last 100 ns of MD trajectories, PCA of β-LG backbones was carried out for each model by using the CPPTRAJ module of AmberTools. The graphical summaries of motions along the first two eigenvectors are shown in porcupine plots using the VMD software version 1.9.3 [35].

### 3.5. Noncovalent Interactions

NCIplot calculations were carried out with a step size of 0.10 to visualize the interacting regions between β-LG and the binding compounds [36,37]. The reduced gradients were rendered as an isosurface in VMD, using an isovalue of 0.3 au.

### 3.6. Binding Free Energy Analysis

The binding free energy between β-LG and EGCG/PIC was obtained from MM/GBSA calculations [38]. A total of 200 snapshots evenly extracted from the last 100 ns of the MD trajectory were used for the calculation of each binding complex. The binding free energy value is equal to the free energy difference between the binding complex (G_complex_) and the sum of β-LG (G_receptor_) and compound (G_ligand_) as follows:

(1) $$∆G_{bind}=G_{complex}-\;(G_{receptor}\;+\;G_{ligand})\;$$

Each of them can be calculated with the equation:

(2) $$∆G_{bind}=\Delta H\;-\;T\Delta S\approx {∆E}_{MM}\;+\;{∆G}_{solv}-T∆S$$

where ΔE_MM_ is the molecular mechanical energy of the gas phase, ΔG_solv_ is the solvation-free energy, and TΔS is the entropy contribution. ΔE_MM_ comprises contributions from electrostatic energy (ΔE_ele_), van der Waals interaction energy (ΔE_vdw_), and internal strain energy (ΔE_int_). Because we adopted the single simulation approach (only simulating the binding complex), ΔE_int_, which comprises bonds, angles, and dihedral energies, will cancel out according to Equation (1) [39]:

(3) $$∆E_{MM}={∆E}_{ele}\;+\;{∆E}_{vdw}\;+\;{∆E}_{int}$$

ΔG_solv_ contains contributions from a polar part (ΔG_GB_) and a nonpolar (ΔG_SA_) part:

(4) $$∆G_{solv}={∆G}_{GB}\;+\;{∆G}_{SA}$$

${∆G}_{GB}$ was estimated by the generalized Born (GB) model with the interior and exterior dielectric constants set to 4 and 80, respectively [40]. The nonpolar solvation terms were calculated according to the LCPO algorithm:

(5) $${∆G}_{SA}=\gamma ∆SASA\;+\;\beta$$

where γ and β were set to 0.0072 kcal∙mol^−1^∙Å^−2^ and 0, respectively [39,41]. Therefore, the binding free energy was calculated as follows:

(6) $${∆G}_{bind}={∆E}_{ele}\;+\;{∆E}_{vdw}\;+\;{∆G}_{GB}\;+\;{∆G}_{SA}-T∆S$$

Based on the extracted snapshots, the entropic contribution (TΔS) was evaluated through normal mode analysis (NMA) [42,43].

## 4. Conclusions

Through intensive molecular docking and molecular dynamics simulations combined with in-depth analyses, the current work characterized the binding features of β-LG with functional compounds including PIC, EGCG, and PLM. Observations on the interaction modes and binding affinities revealed that polyphenols PIC and EGCG may concomitantly bind to the peripheral and central sites, respectively. The central bound PLM showed a synergistic effect on PIC. Key residues that contributed to the binding were identified, and vdW interactions were discovered to play a pivotal role in the binding of β-LG and all compounds.

The major limitation of the current study comes from the research methods of molecular docking and MD simulation. Though three replicas with distinct starting structures for each model were simulated, differences exist between the models and experimental conditions. However, we believe the limitation should hardly influence the conclusion since the investigation was based on the crystal structure of β-LG, and the docking-derived binding sites of compounds are in agreement with the reported experimental results.

Based on our identified interaction modes and key residues that form direct contacts with the binding compounds, site-directed mutagenesis would certainly lead to improved β-LG variants with higher affinities to the target compound. In all, the study here provides pivotal insights into the binding characteristics of β-LG with PIC, EGCG, and PLM, shedding new light on the development of β-LG-based protection and transportation of functional compounds.

## Figures and Tables

**Table 1:** Binding free energies between β-LG and the binding compounds ^a^.

Receptor	Ligand	Energy Component					
		∆E_ele_	∆E_vdW_	∆G_GB_	∆G_SA_	–TΔS	∆G_bind_
β-LG	PIC	−13.66 ± 1.47	−24.28 ± 3.28	13.81 ± 0.94	−3.68 ± 0.12	17.50 ± 11.76	−10.32
β-LG	EGCG	−13.74 ± 3.23	−35.50 ± 3.03	15.76 ± 2.75	−4.91 ± 0.23	21.18 ± 10.27	−17.20
β-LG	PLM	12.36 ± 7.19	−36.38 ± 2.76	−12.82 ± 5.77	−6.23 ± 0.31	23.78 ± 11.07	−19.30
β-LG	PLM	19.47 ± 7.58	−37.04 ± 0.05	−18.87 ± 6.15	−6.16 ± 0.17	23.43 ± 10.49	−19.17
	PIC	−12.84 ± 1.54	−23.33 ± 3.10	13.01 ± 0.94	−3.75 ± 0.09	14.09 ± 10.99	−12.83
β-LG	PLM	15.4 ± 8.84	−35.85 ± 2.84	−15.54 ± 7.15	−6.26 ± 0.21	20.38 ± 11.04	−21.88
	EGCG	−17.07 ± 5.37	−36.93 ± 4.78	19.1 ± 4.25	−5.22 ± 0.20	24.41 ± 11.44	−15.71
β-LG	PIC	−12.85 ± 1.58	−22.91 ± 3.23	12.85 ± 0.99	−3.63 ± 0.11	16.72 ± 10.55	−10.00
	EGCG	−3.95 ± 2.52	−37.32 ± 5.03	7.83 ± 2.14	−5.40 ± 0.40	21.81 ± 11.98	−17.03
β-LG	EGCG	−9.45 ± 4.74	−26.87 ± 6.59	11.73 ± 3.97	−4.09 ± 0.48	22.31 ± 10.25	−6.37
	PIC	−3.71 ± 1.22	−32.83 ± 2.45	6.78 ± 0.81	−4.73 ± 0.12	17.31 ± 9.25	−17.18

^a^ Energies are in kcal∙mol^−1^.

## Data Availability

Data are contained within the article and Supplementary Materials.

## Supplementary Materials

The following supporting information can be downloaded at: https://www.mdpi.com/article/10.3390/molecules29050956/s1. Table S1. Molecular docking results of β-LG and PIC calculated with AutoDock Vina and scored with Dock6. Table S2. Molecular docking results of β-LG and EGCG calculated with AutoDock Vina and scored with Dock6. Table S3. Molecular docking results of β-LG and PLM calculated with AutoDock Vina and scored with Dock6. Table S4. Molecular docking results of β-LG + PIC and EGCG calculated with AutoDock Vina and scored with Dock6. Table S5. Molecular docking results of β-LG + EGCG and PIC calculated with AutoDock Vina and scored with Dock6. Table S6. Detailed information of the constructed models. Figure S1. Superimposition of the best-performed docking conformation of PLM (green) on the crystal configuration (yellow). Figure S2. Initial structures of the β-LG+PIC/EGCG/PLM binary complexes submitted to MD simulations. For PIC and EGCG, three initial docking structures, i.e. replica 1 (orange), replica 2 (blue), and replica 3 (violet), were used in (**a**) and (**b**). For PLM, the crystal configuration was used (**c**). Figure S3. Initial structures for the simulation of the (β-LG + PLM) + PIC/EGCG and (β-LG + PIC/EGCG) + EGCG/PIC tertiary complexes. (**a**–**d**) For PIC and EGCG, three initial docking structures, i.e. replica 1 (orange), replica 2 (blue), and replica 3 (violet), were used. Figure S4. Eigenvalue profiles plotted by the first 30 eigenvectors of the compound bound β-LG in PCA analysis. Figure S5. RMSD profiles of the additional replica MD simulations and the conformational comparison of the equilibrated structures. For clarity, only the β-LG structure in replica 1 was shown, with the compounds in replica 1, replica 2, and replica 3 colored in orange, blue, and violet, respectively. Figure S6. RMSF profiles of β-LG with two compounds bound concomitantly. (**a**–**d**) RMSFs derived from trajectory analysis and the b-factor of crystal structure are colored in blue and black, respectively. Figure S7. RMSD profiles of the additional replica MD simulations and the conformational comparison of the equilibrated structures. For clarity, only the β-LG structure in replica 1 was shown, with the compounds in replica 1, replica 2, and replica 3 colored in orange, blue, and violet, respectively.
