# Supplementary material for: In Silico Investigations on the Synergistic Binding Mechanism of Functional Compounds with Beta-Lactoglobulin
Source: Molecules. 2024 Feb 22;29(5):956. doi: 10.3390/molecules29050956 (PMC10933772; doi:10.3390/molecules29050956)
Supplement: Supplementary file 1 [file molecules-29-00956-s001.zip › molecules-2826306-supplementary.pdf]

In silico investigations on the synergistic binding mechanism of functional compounds with beta-lactoglobulin

Tong Meng,[1] Zhiguo Wang,[2,*] Hao Zhang,[1] Zhen Zhao,[1] Wanlin Huang,[3] Liucheng Xu,[3] Min Liu,[4] Jun Li, [1] and Hui Yan[1,*]

[1]School of Pharmaceutical Sciences, Liaocheng University, Liaocheng, Shandong, 252059, China

[2]Institute of Ageing Research, School of Basic Medical Sciences, Hangzhou Normal University, Hangzhou, Zhejiang Province, 311121, China

[3]School of Basic Medical Sciences, Hangzhou Normal University, Hangzhou, Zhejiang Province, 311121, China

[4]School of Chemistry and Chemical Engineering, Liaocheng University, Liaocheng, Shandong 252059, China

Corresponding authors:

Zhiguo Wang: zhgwang@hznu.edu.cn

Hui Yan: yanhui@lcu.edu.cn

Table S1. Molecular docking results of β-LG and PIC calculated with AutoDock Vina and scored with Dock6.

Mode	Binding energy (kcal/mol)	RMSD (Å)	Amber score (kcal/mol)
1	-7.40	0.00	-2.40
2	-7.30	13.46	13.62
3	-7.20	1.36	-4.91
4	-7.10	1.10	-7.10
5	-7.00	1.15	-3.01
6	-6.70	23.24	-3.19
7	-6.70	3.60	-4.29
8	-6.70	4.41	-4.35
9	-6.60	2.67	-8.71
10	-6.60	4.34	-9.81

Table S2. Molecular docking results of β-LG and EGCG calculated with AutoDock Vina and scored with Dock6.

Mode	Binding energy (kcal/mol)	RMSD (Å)	Amber score (kcal/mol)
1	-9.70	0.00	-8.31
2	-9.20	1.93	-2.97
3	-9.10	1.94	-1.37
4	-8.90	1.97	-8.74
5	-8.90	2.28	-3.62
6	-8.80	2.82	-7.52
7	-8.70	2.29	-2.05
8	-8.60	2.62	-9.08
9	-8.30	1.99	-4.4
10	-8.30	2.62	-9.50

Table S3. Molecular docking results of β-LG and PLM calculated with AutoDock Vina and scored with Dock6.

Mode	Binding energy (kcal/mol)	RMSD (Å)	Amber score (kcal/mol)
1	-6.70	0.00	-23.79
2	-6.00	7.14	-38.31
3	-5.90	6.26	-38.61
4	-5.80	18.69	-18.81
5	-5.60	2.49	-46.31
6	-5.60	1.63	-35.39
7	-5.20	18.84	-13.32
8	-5.20	7.93	-3.19

Table S4. Molecular docking results of β-LG+PIC and EGCG calculated with AutoDock Vina and scored with Dock6.

Mode	Binding energy (kcal/mol)	RMSD (Å)	Amber score (kcal/mol)
1	-5.44	0.00	7.55
2	-5.38	0.15	11.78
3	-5.28	2.21	-12.93
4	-5.14	2.49	-14.57
5	-5.08	2.75	-7.13
6	-4.84	3.10	-10.63
7	-4.81	2.80	2.57
8	-4.67	1.95	-11.83
9	-4.55	3.19	-3.16
10	1.90	2.20	-10.54

Table S5. Molecular docking results of β-LG+EGCG and PIC calculated with AutoDock Vina and scored with Dock6.

Mode	Binding energy (kcal/mol)	RMSD (Å)	Amber score (kcal/mol)
1	-7.56	0.00	-7.51
2	-7.44	2.53	-12.65
3	-7.37	0.32	-7，76
4	-7.29	3.55	-15.12
5	-7.24	3.53	-12.11
6	-7.20	2.73	-20.69
7	-6.68	2.30	3.7
8	-6.57	1.62	5.40
9	-6.56	2.11	13.03
10	-6.43	4.00	-7.23

Table S6. Detailed information of the constructed models.

Model	Number of Na^+	Number of waters	Box volume ($Å^3$)	Concentration of Na^+ (mM)
β-LG+PIC	8	4973	2.05×10^5	64.82
β-LG+EGCG	8	5039	2.06×10^5	64.51
β-LG+PLM	9	4969	2.06×10^5	72.57
(β-LG+PLM)+PIC	9	4982	2.08×10^5	71.88
(β-LG+PLM)+EGCG	9	5087	2.08×10^5	71.88
(β-LG+PIC)+EGCG	8	5008	2.09×10^5	63.58
(β-LG+EGCG)+PIC	8	5062	2.08×10^5	63.89

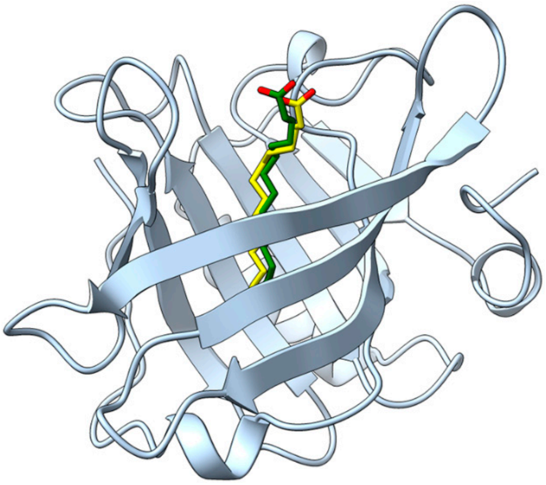

Figure S1. Superimposition of the best-performed docking conformation of PLM (green) on the crystal configuration (yellow).

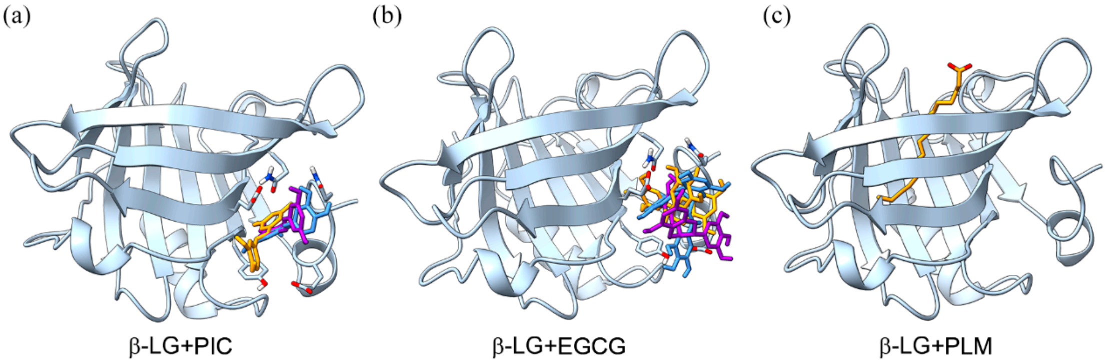

Figure S2. Initial structures of the β-LG+PIC/EGCG/PLM binary complexes submitted to MD simulations. For PIC and EGCG, three initial docking structures, i.e. replica 1 (orange), replica 2 (blue), and replica 3 (violet), were used in (a) and (b). For PLM, the crystal configuration was used (c).

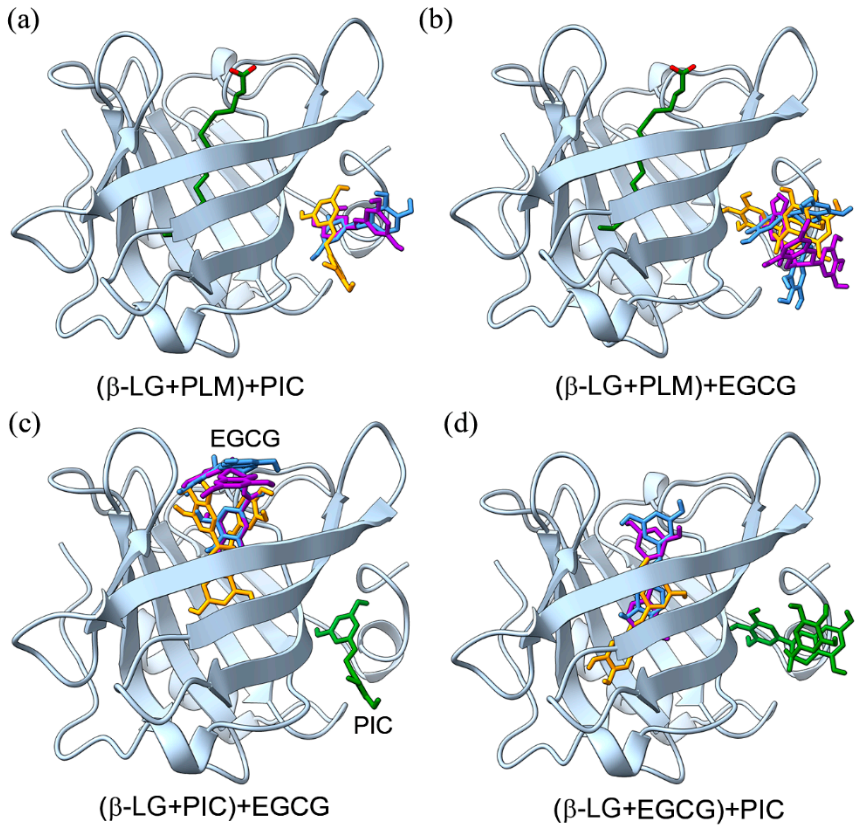

Figure S3. Initial structures for the simulation of the (β-LG+PLM)+PIC/EGCG and (β-LG+PIC/EGCG)+EGCG/PIC tertiary complexes. (a)-(d) For PIC and EGCG, three initial docking structures, i.e. replica 1 (orange), replica 2 (blue), and replica 3 (violet), were used.

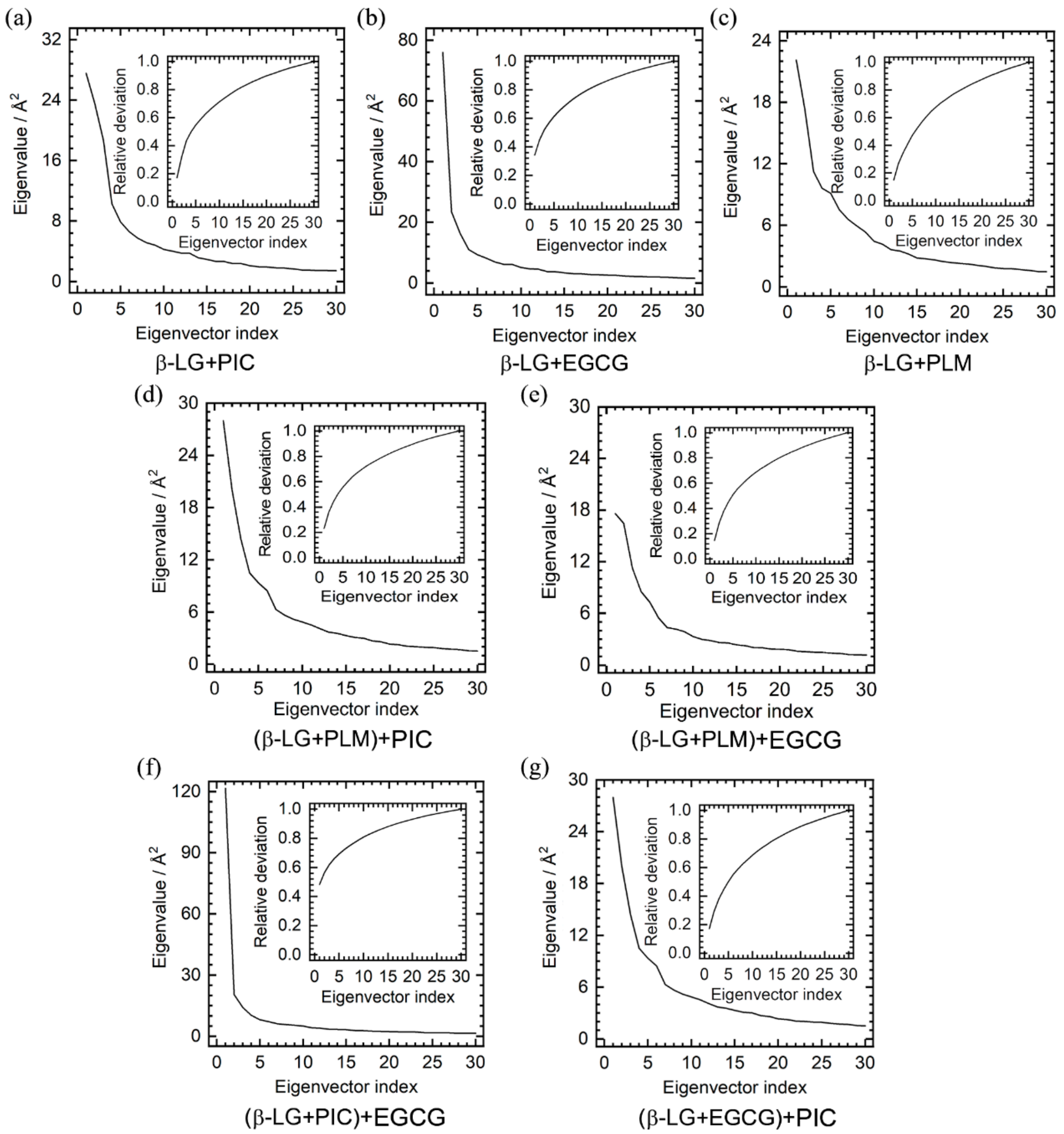

Figure S4 Eigenvalue profiles plotted by the first 30 eigenvectors of the compound bound β-LG in PCA analysis.

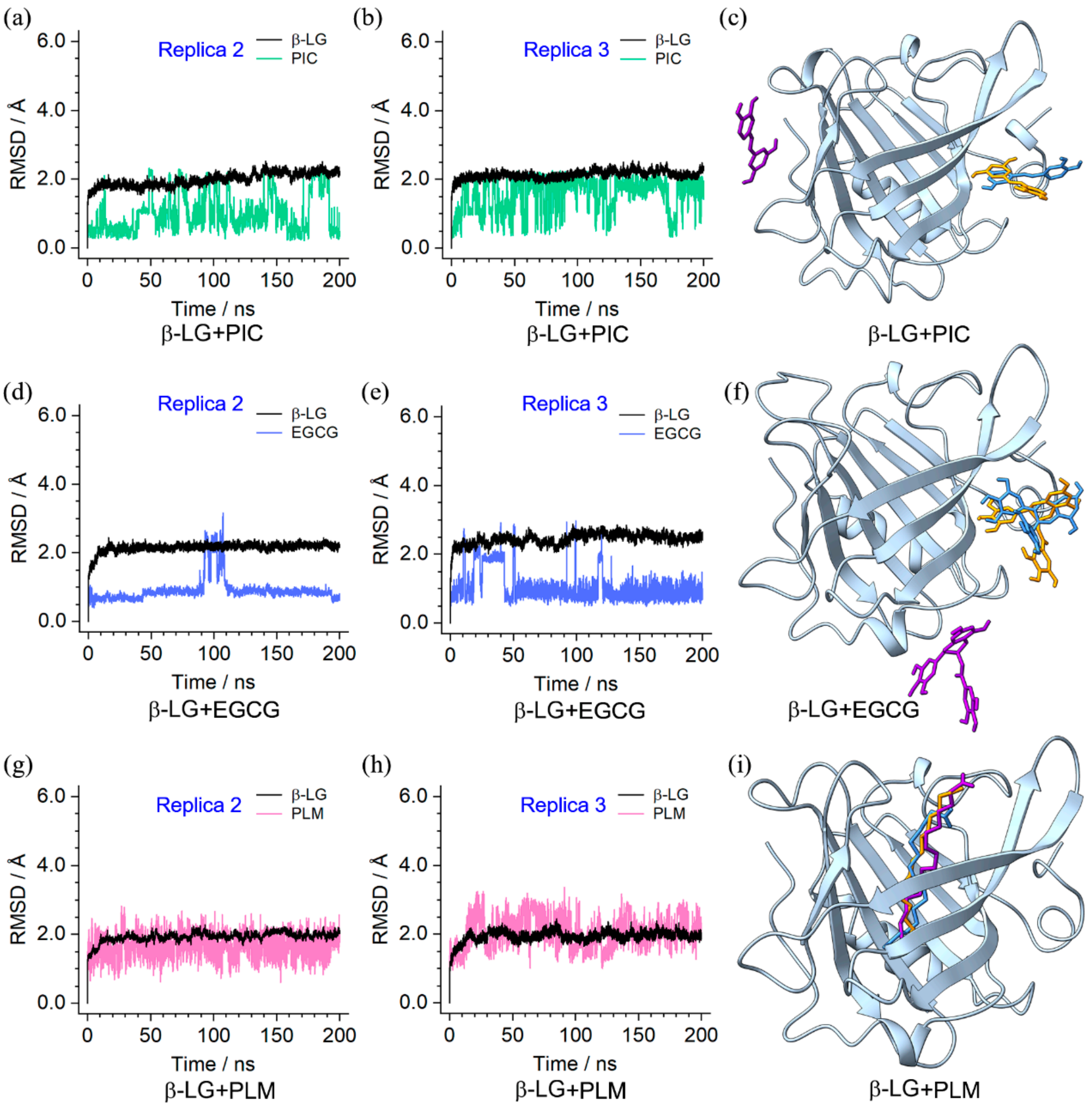

Figure S5. RMSD profiles of the additional replica MD simulations and the conformational comparison of the equilibrated structures. For clarity, only the β-LG structure in replica 1 was shown, with the compounds in replica 1, replica 2, and replica 3 colored in orange, blue, and violet, respectively.

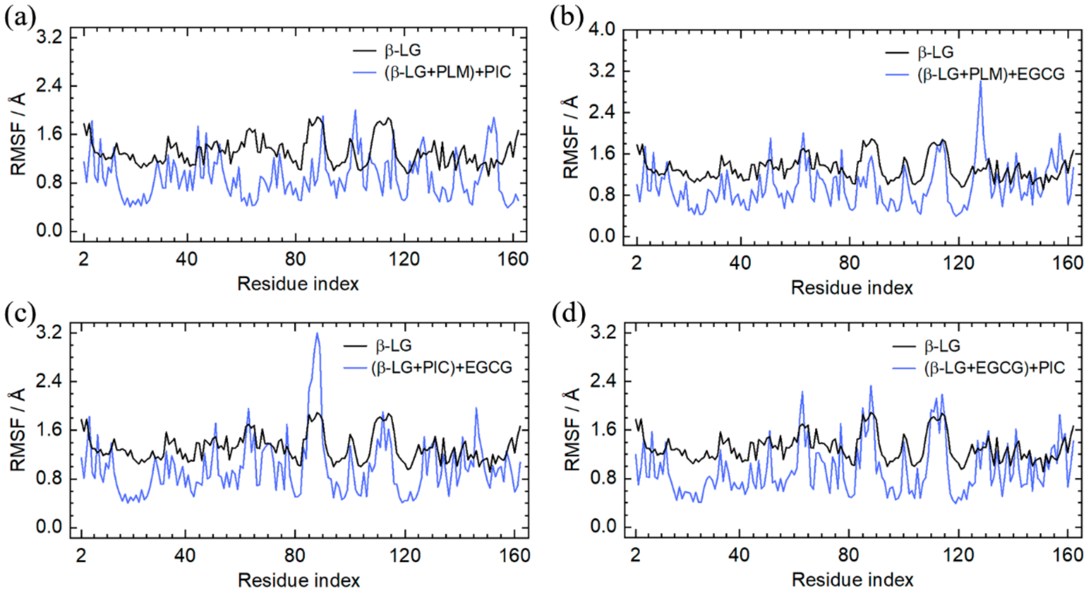

Figure S6 RMSF profiles of β-LG with two compounds bound concomitantly. (a–d) RMSFs derived from trajectory analysis and the b-factor of crystal structure are colored in blue and black, respectively.

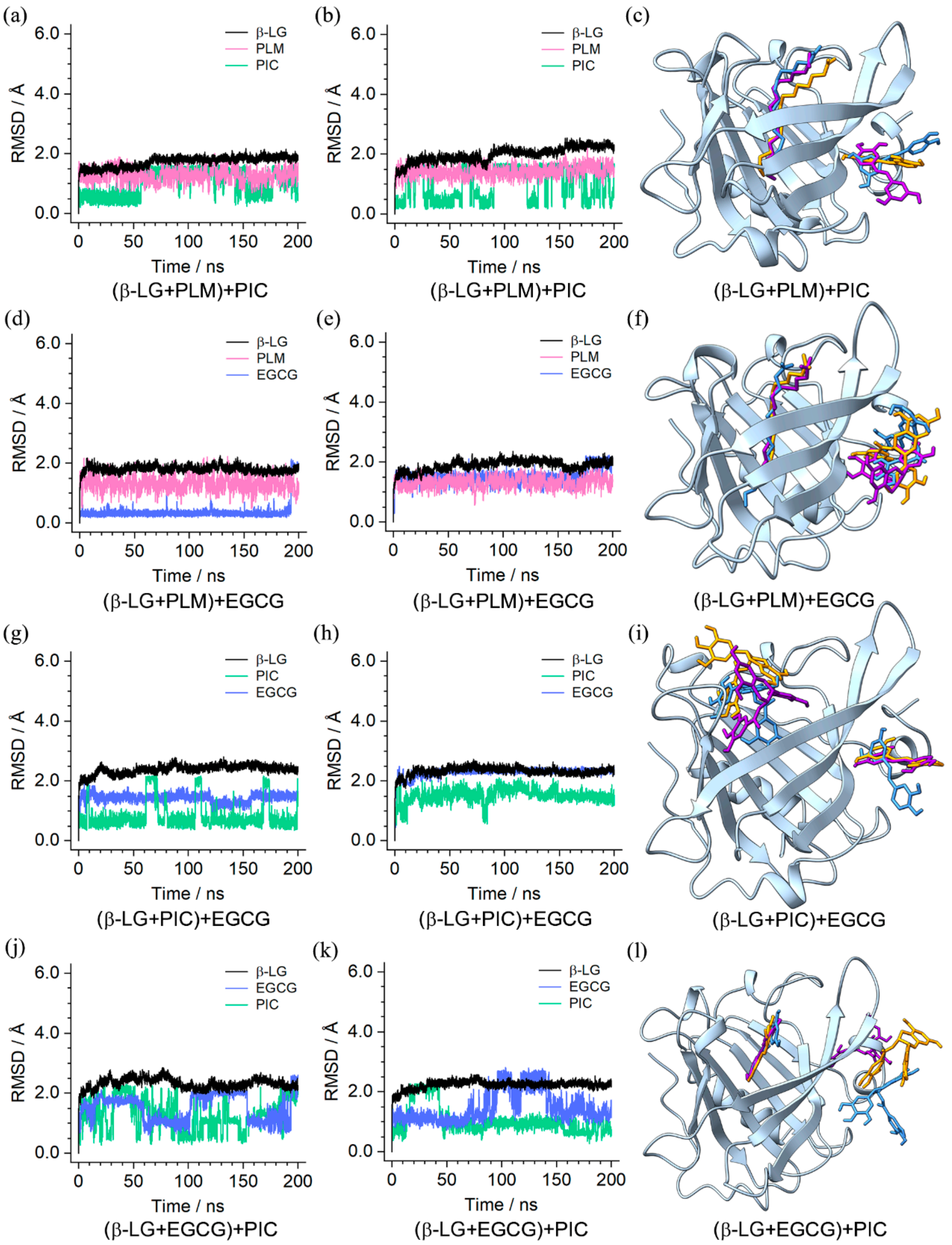

Figure S7. RMSD profiles of the additional replica MD simulations and the conformational comparison of the equilibrated structures. For clarity, only the β-LG structure in replica 1 was shown, with the compounds in replica 1, replica 2, and replica 3 colored in orange, blue, and violet, respectively.
